# Knowledge, application and how about competence? Qualitative assessment of multiple-choice questions for dental students

**DOI:** 10.1080/10872981.2020.1714199

**Published:** 2020-01-14

**Authors:** Mesküre Capan Melser, Verena Steiner-Hofbauer, Bledar Lilaj, Hermann Agis, Anna Knaus, Anita Holzinger

**Affiliations:** a Research Unit for Curriculum Development, Teaching Center, Medical University of Vienna, Vienna, Austria; b University Clinic of Dentistry/Medical University of Vienna, Vienna, Austria; c Department of Conservative Dentistry and Periodontology, School of Dentistry, Medical University of Vienna, Vienna, Austria; d Austrian Cluster for Tissue Regeneration, Vienna, Austria

**Keywords:** Multiple choice questions, NBDE, undergraduate medical education, cognitive level, Moore’s framework

## Abstract

**Background**: Many medical schools train their faculty members to construct high cognitive level multiple choice questions (MCQs) that demand a great deal of analytical and critical thinking, application, and competence. The purpose of this study is to determine the cognitive levels of MCQs by using Moore’s Expanded Outcomes Framework and to understand whether the quality of MCQs has an effect on students’ assessment performance.

**Methods**: Four trained faculty members analysed 100 randomly selected questions developed at the University Clinic of Dentistry (UCD) and 100 questions developed by the National Board of Dental Examinations (NBDE). Moore’s framework was applied to assist the review process.

**Results**: The majority of questions was at the level of declarative knowledge followed by questions at the level of procedural knowledge. The cognitive level of UCD questions from 2002 to 2009 was significantly lower than that of NBDE questions but increased in questions written from 2010 to 2018. The improvement of quality of MCQs had no impact on assessment performance of students.

**Conclusion**: The enhanced cognitive levels of UCD MCQs written 2010–2018 coincides with the implementation of a faculty training program for writing high-ordered MCQs. In addition, this study shows that the use of Moore’s expanded framework is on par with other known taxonomies in supporting educators in writing items and reviewing the process.

**Abbreviations:** MCQs: Multiple Choice Questions; UCD: University Clinic of Dentistry; NBDE: National Board of Dental Examinations

## Background

MCQs are commonly used in assessments at undergraduate and postgraduate levels of medical examinations due to the fact that they are time-efficient, highly reliable and conveniently standardized [1,2]. Well-constructed MCQs allow the evaluation of taxonomically higher-order cognitive skills such as application of knowledge, interpretation or synthesis rather than testing the mere recall of isolated facts [3]. On the other hand, it can be challenging to construct MCQs which evaluate the application of knowledge and students’ competence [4]. In most medical schools faculty members are not trained or only trained in developing basic recall MCQs, which are poorly linked to professional reality [5].

There are numerous frameworks to support the educators’ understanding of the development of learning associated with educational programs. These programs include developmental frameworks and analytical models that address knowledge, skills and attitudes. Some frameworks such as Blooms’ Taxonomy and Millers’ Pyramid are well known and have been used in undergraduate education for creating multiple choice type questions [2,6,7]. There is also Moore’s expanded outcomes framework [8], which is commonly used for the development of new educational activities that enable learners to achieve higher levels of knowledge, competence, and performance (Table 1) [9]. This framework has also been used by Vanderbilt et al. to organize and categorize their medical school MC questions to determine whether the currently used MC questions measure their students’ learning performance [5].

**Table 1.** Moore’s expanded outcomes framework (2009)

Outcomes Framework	Millers’ Framework	Description
Participation **Level 1**		The number of physicians and others who participated in the educational activity
Satisfaction **Level 2**		The degree to which the expectations of the participants about the setting and delivery of the educational activity were met
Learning: Declarative Knowledge **Level 3a**	**Knows**	The degree to which participants state what the educational activity intended them to know
Learning: Procedural Knowledge **Level 3b**	**Knows How**	The degree to which participants state how to do what the educational activity intended them to know how to do
Learning: Competence **Level 4**	**Shows How**	The degree to which participants show in an educational setting how to do what the educational activity intended them to be able to do
Performance **Level 5**	**Does**	The degree to which participants do what the educational activity intended them to be able to do in their practices
Patient Health **Level 6**		The degree to which the health status of patients improves due to changes in the practice behaviour of participants
Community Health **Level 7**		The degree to which the health status of a community of patients changes due to changes in the practice behaviour of participants

The purpose of this study was to analyse the cognitive levels of MCQs used for summative assessment at the end of the fourth year in a clinical undergraduate curriculum at the University Clinic of Dentistry (UCD) at the Medical University of Vienna by using Moore’s expanded outcomes framework because of its accordance with well-known taxonomies (Table 2). The cognitive levels of questions were distinguished as followed: (1) declarative knowledge (recall) questions assessing the acquisition and interpretation of facts (Moore’s Level 3a = Miller’s ‘knows’), (2) Procedural knowledge (application) questions examining the procedural knowledge and/or the knowledge of how to do something (Moore’s Level 3b = Miller’s ‘knows how’) and (3) Competence questions testing to what extent students are able to perform a procedure that has previously been demonstrated in an educational setting (Moore’s Level 4 = Miller’s ‘shows how’. We explicitly included the definition of cognitive levels from other taxonomies such as Bloom’s Taxonomy [10], Millers’ Pyramid and cognitive levels of the National Board of Dental Examinations (NBDE) in order to differentiate the competence questions from application questions in a better way. The main aim of reviewing MCQs was to specify the quality of our MCQs that will measure higher level knowledge as defined by Moore and to understand whether higher quality MCQs that align Moore’s three levels has an impact on students’ assessment performance. Assessment performance is indicated by the grade average.

**Table 2.** Frameworks – From Miller to Bloom to Moore

	Definition of LEVELS		
	Level 1/Level 3a	Level 2/Level 3b	Level 3/Level 4
Moore’s Expanded Outcomes Framework	**Declarative Knowledge**: The acquisition and interpretation of facts	**Procedural Knowledge**: Participants who possess procedural knowledge can describe how to do something but may not be able to actually do it	**Competence**: Participants show in an educational setting how to do what the educational activity intended them to be able to do
Millers’ Pyramid	Knows	Knows How	Shows How
Blooms’ Taxonomy	Knowledge of Learning	Comprehension	Problem-Solving
Anderson & Krathwohl 2001 (revised version of Blooms’ Taxonomy)	**Knowledge & Remembering**: Learner must recall or recognize specific information	**Analysis & Application**: Learner must understand or be able to explain in own words previously learned information and use new information, rules, methods, concepts, principles, and theories	**Synthesis & Evaluation**: Transferring existing knowledge and skills to new situations, understanding a concept’s components and their relationships to each other, and analyse information
Cognitive Levels of National Board of Dental Examination	**Understanding**: Items elicit knowledge of specific facts, terminology, sequences, methodology, principles, theories, and structures in a different context.	**Application**: Items elicit application of specific facts, terminology, sequences, methodology, principles, theories, and structures in a complex manner.	**Reasoning**: Items elicit understanding or the ability to identify and interpret specific data, terminology, sequences, methodology, principles, theories, and structures.

Additionally, we analysed NBDE Part II questions for evaluating the comparability or similarity of cognitive levels of our MCQs. NBDE is conducted in the USA for determining qualifications of dentists who seek licensure to practice dentistry. It assesses the ability to understand important information from basic biomedical and dental sciences as well as the ability to apply such information in a problem-solving context [11]. With regard to development of exam items, a guideline has been published by the Joint Commission on National Dental Examinations to inform dental and academic communities interested in the item development process of National Board Dental and Dental Hygiene Examinations. This guide also intends to pave the way for developing new and current items using the definition of cognitive levels of NBDE to ensure the quality of exam items [12]. As NBDE has a standard item development process and clearly defines cognitive attributes, we analysed the NBDE Part II questions as a reference that are generally used in fourth year of dental education and focus on clinical dentistry.

### Current curriculum

The current dentistry program at the Medical University of Vienna was introduced in 2002, together with the newly integrated curriculum for medical students in the so-called Vienna Medical Curriculum (VCM) (6). VCM of Dentistry degree programme is based on integration of non-clinical and clinical learning: in each learning unit (Module) students learn about structure and function, as well as the most important and most common diseases and therapies. In addition, there are courses called ‘Lines’ which take place throughout the semester. The Lines connect module content with clinical work by focusing on clinical skills. Problem-based learning (PBL) and case-based learning (CBL) supplement other instructional methods. The duration of the Dentistry degree programme is 12 semesters: The first two academic years of this program is partially identical with human medicine and focuses on basic medical sciences. The third year of study is composed of dental-oriented courses and covers three modules such as chewing and locomotor system, oral pathology and internal organs as well as brain, muscles and sensory organs. The dentistry degree programme in 4^th^ year consists of 6 modules and covers areas as paediatric dentistry, cariology, dental restoration, endodontics, periodontology, prophylaxis, restorative dentistry, permanent dentures, prosthetics, surgery and orthodontic followed by a clinical internship of 72 weeks in the last 5^th^ and 6^th^ academic years that focuses on intensive clinical training [13].

Since 2002, a range of different assessment formats are used. Written examinations such as multiple choice questions has been implemented to curriculum to enhance the objectivity and reliability. Continuous assessments in written format or oral contribution take place at the end of each practical training and seminars. Some seminars follow a team-based-learning format. The formative-integrated examinations in MCQs format take place at the end of each winter semester to provide students with feedback on their learning performance. The summative-integrated examination in MCQ format takes place at the end of each academic year. The last summative-integrated examination in MCQ format is integrated at the end of fourth year which contains in total 165 MCQs and is assessing students’ competence and clinical knowledge in subject areas and materials from an entire fourth academic year before entering the dental practice environment which starts in the 5^th^ year and ends at the end of 6^th^ year with oral examination.

### Quality of MCQs

Starting in 2001, the quality of MCQs is evaluated by an interdisciplinary review committee that consists of specialized experts, exam auditors and module coordinators. The committee’s tasks are correcting and formatting and of grammatical errors and detecting ambiguity in the interpretation of questions. Items deemed ambiguous, flawed or irrelevant to the committee were returned to the authoring faculty member with suggestions for improvement. Only questions that were approved by the committee are added into our item bank. With regard to quality assurance of MCQs, training programs are necessary to construct high quality MCQs. Since 2010, a mandatory training program, called ‘Examining clinical competence of medical students with multiple-choice questions’, has been integrated in advanced medical education courses for junior faculty members. Currently, this training programme is also referring to faculty tenure as an optional workshop to qualify for permanent position. Our MCQs are written by both trained and non-trained faculty members using Millers’ pyramid and internal quality criteria such as reliability, validity and discrimination index. Point biserial correlation is used to determine the discrimination index of items. The content validity of assessment has been increased by developing blueprint process for each summative-integrated assessments. MCQs are constructed to test knowledge and definitions of facts (‘knows’), but also require reaching a conclusion, formulating a prognosis, or selecting options for action (‘knows how’).

### Study objectives

The objectives of this study were (1) to determine whether MCQs from UCD have an international comparable cognitive level (UCD vs. NBDE); (2) to address the development of MCQs from 2002 to 2018; (3) to analyse the impact of different subject areas on constructing high ordered MCQs that promote and measure competency and critical thinking ability of students; (4) to evaluate the role of item difficulty – an important parameter for increasing the MCQs’ quality – on the distribution of cognitive level of our MCQs.

## Methods

### Selection of MCQs

In total, two hundred MCQs (100 UCD, 100 NBDE) were evaluated. For setting up MCQs pools from our item bank for the fourth year summative-integrated examination, which contains 4933 approved MCQs, we used the following criteria: (1) MCQs that had been used only one time; (2) 50% of MCQs dated from 2002 to 2009 (pool 1) and 50% from 2010 to 2018 (pool 2) to evaluate the distribution of the cognitive levels of MCQs over the course of time.

Fifty UCD-MCQs from each pool were selected using a random number generator. Using these particular questions, we wanted to determine if we are able to develop high levels of MCQs for our students to further examine their critical and analytical thinking skills.

100 of the 200 NBDE Part II questions were chosen randomly from the officially released item test package dating approximately from 2000 to 2008. A request to the American Dental Association for questions from 2009 to 2018 for study purposes was not honoured, and the questions have not been provided. Therefore, we were just able to analyse 100 MCQs of NBDE and compared with our 50 MCQs from year 2002 to 2009.

### Reviewers

All reviewers, who participated this study, had participated in faculty development program directed towards the construction of MCQs. The two faculty members with clinical experience in dentistry assessed and interpreted the questions at the clinical level. They have both had several years’ experience on constructing of MCQs. Another faculty member was a regular item writer with research experience in the field of theoretical dentistry. The last reviewer had several years of experience in preparing exams and selecting exam questions in the dental field.

### Evaluation of MCQs

**Figure 1.**
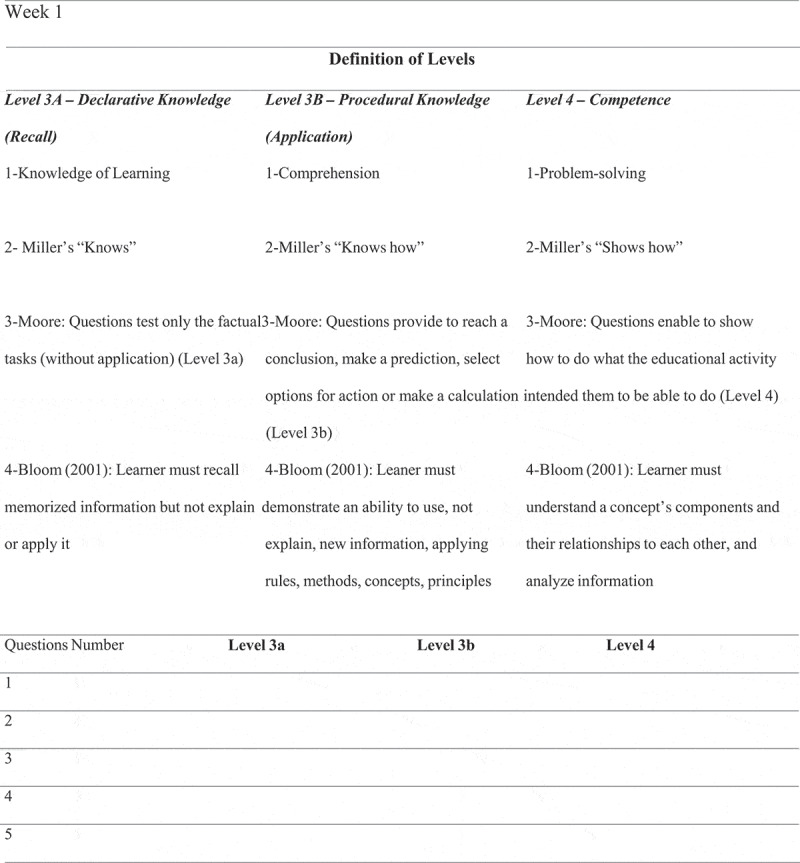
Example of coding from (insert after ‘Evaluation of MCQs’ in methods)

MCQs were analysed by four faculty members with expertise in different fields. The reviewers received 25 questions together with the coding form (Figure 1) once every 2 weeks. After completion of individually coding of each question the reviewers met to discuss and compare their results under supervision of the lead author and one investigator. Each meeting was scheduled for 1 h and the review team met twice per month over the course of a 4-month period. Each reviewer explained why he or she had selected the code (recall or application or competence) for the question and consensus was reached. Inter-rater reliability was 81%. The lead author kept the master copy of the final decisions.

### Statistical analysis

Sample size was calculated by performing a power analysis with power of 0.8, a significance level of 0.05 for a medium effect size according to Cohen [14]. All data were analysed in SPSS 24.0. The level of significance was set at 0.05. Frequency data were calculated to determine the value of the data by using Chi-square test. To avoid potential problems with low expected cell frequencies, we did not compare the resulting test statistic to the usual tabulated value, but to the empirical distribution from B = 100,000 tables created using Monte Carlo simulation [15,16]. The percentage of exam questions were analysed according to declarative knowledge (Level 3a), procedural knowledge (Level 3b), or competence (Level 4).

## Results

### UCD (2002-2009) versus NBDE (2000-2008)

In total, 150 MCQs (50 UCD, 100 NBDE) were analysed. Of 50 MCQs from UCD, 94% questions were categorized as Level 3a, and 6% as Level 3b. On the other hand, the distribution of MCQs from NBDE was as follows: 73% Level 3a, 26% Level 3b and 1% Level 4. According to this data, there was a significant difference (χ^2^ = 9.234; p = 0.005) between the distribution of cognitive levels of NBDE questions and UCD questions (Table 3).

**Table 3.** Comparison of cognitive levels of UCD questions with cognitive levels of NBDE questions

Cognitive Levels	Number of UCD Questions	Number of NBDE Questions	Total Number of MCQs
Comparison of UCD questions (2002–2009) with NBDE questions (2000–2008)			
Level 3a	47	73	120
Level 3b	3	26	29
Level 4	0	1	1
	50	100	150

*χ2 = 9.234

**P-value = 0.005

There was a significant difference between the cognitive levels of UCD and NBDE questions.

### MCQs of UCD 2002-2009 versus MCQs of UCD 2010-2018

Fifty MCQs from the years 2002 to 2009 and 50 MCQs from the years 2010 to 2018 were analysed (Table 4). The older MCQs consisted of 94% of Level 3a (knowledge) questions and 6% of application questions (Level 3b). In the course of time, Level 3a questions have decreased and level 3b questions were increased. The ratio was 64% of Level 3a to 36% of Level 3b questions. One question from 2010 to 2018 was also determined as competence question. (χ^2^ = 13.648; p = 0.001).

**Table 4.** Comparison of cognitive levels of MCQs (2002–2009) with MCQs (2010–2018)

Cognitive Levels	Number of MCQs 2002-2009	Number of MCQs 2010-2018	Total Number of MCQs
Development of UCD questions overtime			
Level 3a	47	32	79
Level 3b	3	17	20
Level 4	0	1	1
	50	50	100

*χ 2 (1, N = 150) = 0.004972

**P-value < .05. There was a significant difference between the cognitive levels of UCD and NBDE questions.

### Assessment performance of students

The results showed that the quality of MCQs had no impact on students’ exam performance. The grade average was 3.3 from 2002 to 2009 and from 2010 to 2018.

### Various specialist fields

To understand the role of specific fields on the distribution of cognitive levels of MCQs, the questions were analysed in terms of their disciplines. The data showed more than 50% of recall questions in all fields. Similar distribution of knowledge questions (59%) and application questions (41%) was seen in the field of prosthetic fundaments and removable prosthetic. In the field of cardiology and orthodontics, the number of application-oriented MCQs increased to up to 22%. The analysis also showed 94% knowledge questions in the field of dental surgery (Table 5). Only one competence question was determined in each of the examinations (UCD/NBDE). In accordance with these results, there was no significant association between subject areas and cognitive levels of MCQs (χ^2^ = 13.021; p = 0.188).

**Table 5.** The role of various subject areas on cognitive levels of MCQs

	Subject Areas						
	Cariology; Dental restoration; Endodontics n – (%)	Periodontology; Prophylaxis n – (%)	Restorative dentistry; Permanent dentures n – (%)	Prosthetic fundaments; Removable prosthetics n – (%)	Surgery n – (%)	Orthodontic n – (%)	Total MCQs n
Distribution of the cognitive levels of UCD questions by subject areas							
Level 3a	13 – (72)	6 – (86)	22 – (88)	10 – (59)	16 – (94)	12 – (75)	79
Level 3b	4 – (22)	1 – (14)	3 – (12)	7 – (41)	1 – (6)	4 – (25)	20
Level 4	1 – (6)	0	0	0	0	0	1
							100

* χ2 = 13.021

**P-value = 0.188

There was no significant associate between cognitive levels and subject area.

### Difficulty levels of MCQs

We determined the role of item difficulty – an important parameter for increasing the MCQs’ quality – on the distribution of cognitive level of our MCQs. Difficulty index (DIFI) is one of the most commonly used statistic parameter for item analysis, and is obtained by dividing the number of students who answered the item correctly by the total number of students who answered that item, thus ranging between 0.0 and 1.0 [17–19]. 82% MCQs from UCD had an adequate level of difficulty between 0.4 and 0.9 (moderately difficult to moderately easy). There was no significant difference between old and new MCQs with regard to ‘item difficulty’ (χ^2^ = 7.278; p = 0.534), and the data were not able to show the impact of difficulty of items on the distribution of the cognitive level of MCQs (Table 6).

**Table 6.** Association between cognitive levels of MCQs with items’ difficulty level

	Difficulty Level of MCQs									
	0.1–0.2	0.2–0.3	0.3–0.4	0.4–0.5	0.5–0.6	0.6–0.7	0.7–0.8	0.8–0.9	0.9–1	
	n	n	n	n	n	n	n	n	n	Total
Distribution of the cognitive levels of UCD questions by difficulty level										
Level 3a	2	3	5	7	17	16	16	10	3	79
Level 3b	0	2	2	2	5	1	2	5	1	20
Level 4	0	0	0	0	1	0	0	0	0	1
										100

*χ2 = 7.278

**P-value = 0.534

There was no significant associate between cognitive levels and difficulty level of questions.

## Discussion

In this study, we evaluated the cognitive levels of MCQs used for summative assessment after the fourth year in a clinical undergraduate curriculum at the UCD by using Moore’s expanded outcomes framework. We analysed whether our currently used MCQs were assessing our students’ abilities at the procedural knowledge level and competence level rather than just at the level of declarative knowledge. We demonstrated that the construction of MCQs at the UCD has improved over time and the vast majority of knowledge questions have been replaced by items measuring procedural knowledge of undergraduate dental students.

The reasons for this could be first, the amount of MCQs promoting longer-term retention of knowledge has increased since a training program has been implemented into advance educational activities for junior faculty members. Second, some faculty members who developing high quality questions may be more interested in constructing questions that measure the application of knowledge and clinical competence of examinees than others. The third reason could be that crafting declarative knowledge questions has already been used up by our item writers therefore they now constructing MCQs that explore a deeper understanding of basic science content which demand the use of higher-order thinking skills.

Previous studies have indicated, that low cognitive level MCQs are mostly written by untrained item writers and constructing MCQs is a creative act that can be mastered through extensive and critically supervised practice [20,21]. After having attended training programs, item writers tend to create adequate-quality and high cognitive level questions [22]. Therefore, the availability of formal training programs is an important step to improve the quality of MCQs and to assess high ordered thinking skills of medical students in undergraduate education.

On the other hand, writing MCQs with high cognitive levels requires a multistep preparation process. An interdisciplinary review committee is one of the critical steps in construction and writing of items. Wallach et al. demonstrated that reading and discussing questions in a committee review process improves not just the quality of questions but also increases the knowledge and information of item writers between various disciplines [23]. As the item writers generally have little motivation to write MCQs that evaluate application of knowledge, synthesis and interpretation of clinical knowledge, the importance of the interdisciplinary review process cannot be overestimated. Such a review process was instituted in Vienna together with a major curriculum reform in 2001 and refined in subsequent years.

Nevertheless, it is also important to ask if it is necessary to assess medical students’ knowledge at the competence level by using MCQ format. Several other methods have been developed and implemented over the time that have focused on clinical skills like taking a history from a patient and performing a physical examination, communication skills, procedural skills, and professionalism such as OSCE. In Miller’s model, written examinations measure what student ‘knows’ or ‘knows how’, and OSCE is a tool for assessing ‘shows how’ [7]. In Moore’s model, the clinical assessment ‘shows how’ is integrated into ‘Level of Learning’ together with ‘knows’ and ‘knows how’ to show that ‘competence’, which measures the critical thinking ability of students, should be implemented in the early stage of medical education through developing higher cognitive level multiple choice questions. Moores’ Framework is used far too little in undergraduate medical activities comparison to Blooms’ taxonomy or Miller’s pyramid. With this in mind, this study points out that Moore’s framework is equally well suited as Blooms’ taxonomy and Miller’s pyramid to support educators in constructing high-quality MCQs.

## Limitations

One limitation of our study is the small number of MCQs that were analysed, which also effects on evaluating the power of ‘difficulty of items and specific fields’ on cognitive levels. Another limitation is the lack of availability of officially published NBDE questions from 2009 to 2018. Therefore, we were not able to compare our newly written MCQs which might have similar amount of high level questions. In spite of the abovementioned limitations, our study results show that training and internal review process enhance the development of higher quality multiple-choice questions that align with the Moore’s framework of Level 3b and Level 4.

## Conclusion

Written examinations are capable of assessing application of knowledge and clinical competence. We observe that the items that require the student to think analytically and to apply their knowledge to practically relevant problems have been increased over time. In order to successfully enhance the quality of MCQs to reach the higher international level, we highly recommend the implementation of Moore’s expanded framework for writing and reviewing items as a first step.

## Data Availability

The questions used during this study are available from the corresponding author on reasonable request.
